# Defining Tumor Microenvironment as a Possible Target for Effective GEP-NENs Immunotherapy—A Systematic Review

**DOI:** 10.3390/cancers15215232

**Published:** 2023-10-31

**Authors:** Paulina Chmiel, Paulina Rychcik-Pazyrska, Rafał Stec

**Affiliations:** Department of Oncology, Medical University of Warsaw, 02-097 Warsaw, Polanddrrafals@wp.pl (R.S.)

**Keywords:** neuroendocrine tumors, immunotherapy, PD-1/PD-L1, TME, GEP-NETs, GEP-NECs

## Abstract

**Simple Summary:**

Therapeutic options for GEP-NETs are very limited, and at the same time, the incidence of these cancers is increasing. The evidence has shown that multiple neoplasms can be successfully treated with immune checkpoint inhibitors such as PD-1/PD-L1 inhibitors. Crucial to determining a patient’s response to immunotherapy is access to the tumor microenvironment. Currently, there is no consistency regarding the future role of TME and immunotherapy in GEP-NETs. Thus, this review points to the solution in selecting a concrete patient group that, based on tumor features can benefit from immunotherapy.

**Abstract:**

Neuroendocrine neoplasms (NENs) are a heterogenous and recurrent group of malignancies originating from neuroendocrine secretory cells diffused on all parts of the human body. Gastro-entero-pancreatic neuroendocrine tumors (GEP-NETs) account for most NENs. Considering the abundance of possible origins, locations, and tumor specifications, there is still no consensus about optimal treatment options for these neoplasms. In light of the escalating immunotherapeutic approaches, it is crucial to define indications for such therapy in GEP-NETs. Bearing in mind the significance of pathophysiological mechanisms and tumor microenvironment (TME) impact on carcinogenesis, defining TME structure and correlation with the immune system in GEP-NETs appears essential. This paper aimed to assess the characterization of the tumor immune microenvironment for a better understanding of the possible therapeutic options in GEP-NETS. The authors performed a systematic review, extracting papers from the PubMed, Web of Science, and Scopus databases according to the Preferred Reporting Items for Systematic Reviews and Meta-Analyses (PRISMA) guidelines. Among 3800 articles identified through database searching, 292 were assessed for eligibility. Ultimately, 28 articles were included in the qualitative synthesis. This paper sums up the research on the immune cell infiltrates, immune checkpoint expression, cytokine profile, neoangiogenesis, and microbiome in the TME of GEP-NETs.

## 1. Introduction

**Figure 1:**
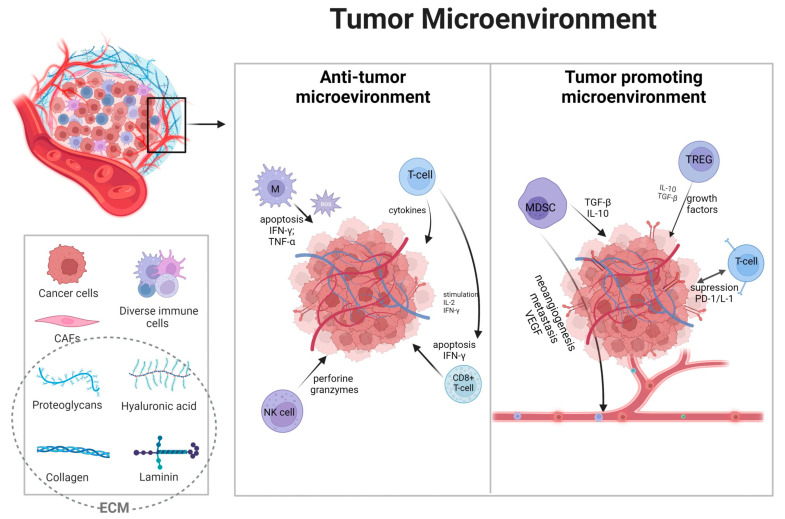
Overview of the tumor microenvironment and its functions. Broadly, immune cells in the microenvironment can be divided into suppressing tumor cells or promoting cancer genesis [24]. Depending on the type, immune cells, grading, and many other factors, tumors can be “hot” with the accumulation of proinflammatory factors or “cold” with poor immune responses [25]. Solid tumors above tumor cells consist of endothelial cells, fibroblast cells, and many innate and adaptive immune cells, with extracellular matrix (ECM) and extracellular factors such as cytokines [26]. Immune cells are essential components, often deciding about therapeutic responses [27]. The main immune cells compromise tumor-associated macrophages (TAMs), myeloid-derived suppressor cells (MDSCs), regulatory T (Treg) cells, and tumor-infiltrating leukocytes (TILs) such as cytotoxic CD8+ T-cells, CD4+ T-cells, and natural killer (NK) cells [28,29]. These cells present complex dependencies with one another, inducing metabolic effects such as hypoxia and neoangiogenesis leading to tumor progression and resistance to therapy [30,31,32]. Furthermore, the basis of communication lies in immune checkpoints such as programmed cell death-1/ligand (PD-1/PD-L1) and cytotoxic T-lymphocyte-associated protein-4 (CTLA-4), expressed both on immune and cancer cells [33,34,35]. Anti-tumor cells, primarily TILs, NK cells, and TAMs, are responsible for killing cancer cells by granule exocytosis and apoptosis induction, secreting multiple pro-inflammatory cytokines (such as IFN-γ, TNF, IL-6) [36,37]. Moreover, NK cells can induce apoptosis by releasing perforin and granzymes [38]. TAMs population is responsible for reactive oxygen species and their influence on tumor regression [39,40]. MDCS and Tregs are key players in immune tolerance [41]. In TME, Tregs infiltration is associated with a worse prognosis, as they prevented effective responses of effector cells [42,43]. In conclusion, the extensive landscape of TME cells is still not thoroughly analyzed, and further research is needed for the putative application of immunotherapy. Created with biorender.com (accessed on 23 July 2023).

Neuroendocrine tumors (NETs) constitute a heterogeneous group of neoplasms arising from the diffuse neuroendocrine system, this includes sites such as the respiratory tract, thyroid, skin, breast, and urogenital system [1]. The most prevalent sites of origin include the small bowel, followed by the lungs and pancreas, while 11–22% of all NETs are defined as unknown primary origin [2]. Gastro-entero-pancreatic neuroendocrine tumors (GEP-NETs) include carcinoid tumors of the gastrointestinal tract and pancreatic NETs (panNETs) [3]. They can be hormonally functioning or nonfunctioning tumors, and based on their site of origin, they can have completely different clinical features [4]. Recently, the 2019 WHO classification, based on the previous 2010 and 2017 WHO reports, established the importance of classification in consideration of the primary site, the morphological differentiation, and the grading [5]. Accordingly, GEP-NETs are grouped into three categories by proliferation index Ki-67. Notably, the new G3 category has been described with Ki-67 > 20% and well-differentiated morphology, in contrast to neuroendocrine carcinomas (NECs) with Ki-67 > 20% and poor differentiation [3]. In recent years, the incidence of GEP-NETs has been constantly increasing from 1.09 per 100,000 in 1973 to 6.98 per 100,000 in 2012, which makes them the second most common digestive cancer [6,7]. GEP-NETs are moderately rare tumors and the diagnosis often comes at advanced stages of the disease, excluding hormonally active tumors with characteristic clinical presentation and a rather quick diagnosis [8]. The diagnosis can be based on clinical presentation with 5-hydroxyindoleacetic acid (5-HIAA) assessment and conventional or functional imaging [9,10]. Computed tomography (CT) constitutes the basic radiological method for NET imaging; other options are magnetic resonance imaging (MRI), endoscopic ultrasound (EUS), and novel strategies such as 68Ga/64Cu-DOTA-somatostatin analog (SSA) positron emission tomography (PET) in combination with CT (PET-CT) [11,12,13,14,15]. Surgery is the cornerstone of treatment for the local or locoregional disease in NET G1 and G2. Furthermore, surgery can be effective also in metastatic disease [16]. Aggressive NECs are commonly treated with systematic platin-based chemotherapy [17]. The last decade brought novel strategies in advanced tumors such as somatostatin analogs (SSAs), mTOR inhibitor (everolimus), tyrosine kinase inhibitor (sunitinib), and peptide receptor radionuclide therapy (PRRT) [18]. Based on two clinical trials with great efficacy and good long-term tolerability, SSAs are established as first-line therapy in some metastatic GEP-NETs [19,20]. Everolimus and sunitinib (the only TKI approved in panNETs) showed similar efficacy in clinical trials [21,22]. Despite undeniable success in managing GEP-NETs, objective remission rates are low, the evidence of long survival in advanced tumors is poor, and clinical trials for many other therapeutic options are lacking. Even in the heydays of immunotherapy, it has still an unclear and fledgling role in GEP-NETs [23]. Concerning the potential use of immunotherapy in this indication, it is crucial to strictly acknowledge the immunological functions of TME in GEP-NETs (Figure 1). Thus, there is an important need to improve our ability to identify patients most likely to benefit from specific therapies in NETs.

## 2. Materials and Methods

**Figure 2:**
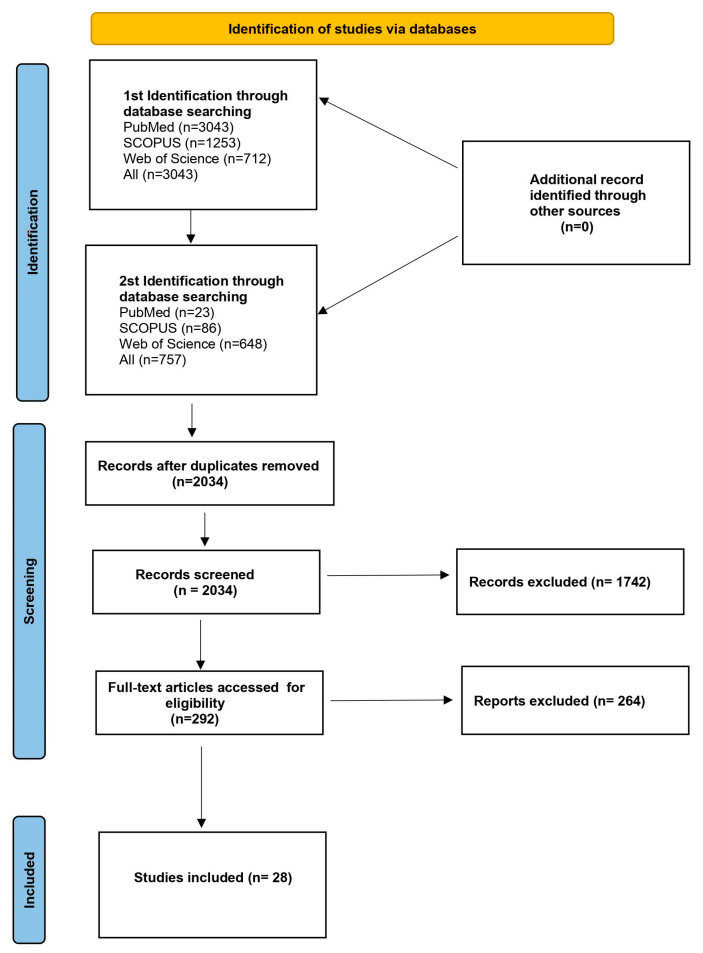
Flowchart presenting the process of article selection, according to Preferred Reporting Items for Systematic Review and Meta-Analyses (PRISMA) guidelines.

The objective of this paper was to conduct a review of the available literature regarding the tumor microenvironment and its clinical implications in neuroendocrine tumors. Classification of the knowledge concerning the NENs TME is crucial for future retrospective and prospective studies. The pattern of the patients and the tumor features must be analyzed to determine the target group for the new therapeutic option in this indication. We followed the Preferred Reporting Items for Systematic Reviews and Meta-analyses (PRISMA) statement guidelines to design, analyze, and report our findings [44]. The systematic review followed the recommendations of the Preferred Reporting Items for Systematic Reviews and Meta-Analyses (PRISMA). The protocol has not been registered. A systematic literature review of the PubMed, Web of Science, and Scopus databases was performed by two identifications in March 2023. The first identification included the search strategy as follows: neuroendocrine tumors OR neoplasms AND immunotherapy OR microenvironment; 3043 articles were found. As we are aware of the heterogeneity of this group of neoplasms in the second identification, we decided to further narrow our field of interest. We continued as the second identification with the use of the following search string: GEP-NENs OR GEP-NETs OR GEP-NECs AND immunotherapy OR microenvironment; 757 articles were found. Eventually, after removing duplicate papers and excluding articles that did not meet the inclusion criteria, 28 articles were included in a qualitative synthesis. The literature search included both human and animal studies. There were no restrictions regarding the year of publication. The authors chose only articles in English. A detailed search strategy is presented in Figure 2. Additional papers were identified by a manual search of the references from the eligible articles.

The inclusion criteria were as follows: (1) the tumor microenvironment was analyzed among gastrointestinal and pancreatic neuroendocrine neoplasms; (2) tumor microenvironment cells had a detailed description, and specific TME molecules/structures were included in the studies; (3) the prognostic values of the microenvironment features were included in the studies; (4) only original research was included. Studies were excluded if they were clinical trials evaluating drugs/medical interventions among patients with GEP-NENs or if the aim of the study was not specifically focused on immune microenvironment features. All studies evaluating neuroendocrine neoplasms outside the gastrointestinal tract were also excluded. Furthermore, any review articles were excluded.

The search results were reviewed by 2 independent researchers (PC and PRP) for potentially eligible studies. The full-text article was searched when there was any statement in the abstract on GEP-NENs microenvironment or immunotherapy. Review articles were also checked in full and references from any full-text articles were cross-checked to ensure inclusion in this review. Disagreements over the eligibility of an article were resolved by consensus.

## 3. Results

A total of 3800 articles were established based on the search terms given above, among which 1766 duplicated records were removed. After the initial screening, 1742 articles not relevant to our study were removed. In total, 292 studies were eligible for a full-text assessment, of which 264 were removed because they did not meet the criteria of inclusion. In the end, 28 studies were included in this review. The time range of the published articles was 2014–2022, and the studies were both prospective and retrospective. Table 1 summarizes the main features of the selected studies, and Table 2 summarizes the key findings of the selected studies.

### 3.1. Immune Checkpoints

Immuno-oncology is a complex field of many mechanisms that play a key role in cancer treatment, genesis, progression, and metastasis. To this day, multiple potential targets have been introduced in this topic. Starting from the PD-1/PD-L1 and CTLA-4 to the newly introduced LAG-3, B7-H3, or VISTA [73,74]. The suppressive functions of immune checkpoints in TME depend on ligand-induced signaling, which is often dysregulated within tumor cells. Discussing all the potential immunotherapy targets is beyond the scope of this paper; however, the foundation of the present approach is mainly PD-1/PD-L1 and CTLA-4, both commonly seen on activated immune system cells, especially activated T-cells [75]. CTLA-4, primarily expressed by T cells, prevents an overactive immune system in the early phase by binding to CD80/CD86 and inhibiting the stimulatory signal of T-cell proliferation provided by CD28 ligand [76,77] In contrast, the binding of PD-1 with its ligand is responsible for the phosphorylation of multiple tyrosine kinases in T-cells and, due to complex signaling pathways, including the activation of Src homology region 2 domain-containing phosphatase (SHP-2), leads to the phosphorylation of downstream signaling of TCR and CD28 [78]. Not only early stages of T-cell activation are dysregulated but also the presentation of antigens is ineffective [79]. Cancer cells, by hijacking the PD-1/PD-L1 pathway, escape surveillance; it is no different in neuroendocrine tumors [80]. Regardless of these promising results, the response rate of PD-1/PD-L1 inhibitors in overall patients is disappointing, which limits the application in clinical practice [81]. Nonetheless, clinical trials have shown the effectiveness of immune checkpoint inhibitors in this indication [82]. Thus, the in-depth analysis of the expression of these molecules in the tumor microenvironment can be crucial for specifying a group of patients that can potentially benefit from immunotherapy. Some features in TME can correlate with answers to immunotherapy, such as checkpoint inhibitors expression, infiltration of TILs, mutation burden, or mismatch-repair (MMR) deficiency [83]. Most of the analyzed studies confirmed the expression of PD-1 and PD-L1 in TME. PD-L1 was mostly expressed by tumor cells, while PD-1 could be observed in both tumor cells and immune infiltrates [45,46]. Furthermore, based on the current research, there is a strong correlation between GEP-NENs grade and progression and PD-L1 expression [47,53]. Simultaneously, it has been stated that PD-L1 is the best predictive marker for a good response to PD-1/PD-L1 blockade therapy [84]. However, a meta-analysis from eight randomized controlled trials stated that axis expression status alone is insufficient in determining which patients should benefit from immunotherapy [85]. Therefore, it seems reasonable to discuss the potential PD-1/PD-L1 expression status in correlation with other factors.

The analysis detected the expression of PD-1/PD-L1 in 1% and 6% of tumor samples, accordingly, and in 8% of peritumoral tissue samples. PD-L1 was expressed mainly in the cell membrane. The main correlation found was between PD-1 expression and higher disease stage and metastases [45]. Furthermore, the analysis of peripheral blood mononuclear cells (PBMC) found that in patients with the progressive disease, there were increased levels of CD3+ PD-1+, CD3+ CD4+ PD-1+, and CD3+ CD4+ CD25+ FOXP3+ PD-1+ cells [45]. In the other study, GEP-NETs showed adaptive immune resistance dependent on CD3+ cells and PD-L1 expression. The expression of PD-L1 on tumor cells varied from 39% in jejunal tumors to 70% in duodenal samples, while PD-1 was not found on the tumor cells. One-third of tumors had no significant immune response [46]. The results of [47] showed that the expression of immune checkpoints in high-grade G3 GEP-NETs differed from the previously mentioned studies. The expression of PD-1/PD-L1 was, respectively, 62% and 38%. Furthermore, tumors with higher expression of PD-L1 and intense PD-1+ CD8+ immune cell infiltration showed the most favorable median OS. The expression of PD-L1 is common in GEP-NENs and increases with grading; 73% of samples were PD-L1 positive. Furthermore, the correlation was observed between PD-L1 expression and somatostatin receptors [53]. The clinical trials of various neoplasms documented significantly better responses among patients with higher PD-1/PD-L1 expression. In the Keynote-052 trial with urothelial cancer, expression above 10% showed a higher objective response rate than the subgroup with PD-L1 expression below 1% [86]. Thus, of the studies considering GEP-NETs showing expression of these molecules, their methodology is inconsistent, and conclusions should be drawn very carefully. However, the analysis of the research allows us to establish a correlation between grade and possible PD-1/PD-L1 expression.

Other reviewed studies about PD-1/PD-L1 expression can be divided into two heterogeneous groups, with detailed results listed in Table 2. The first group showed low expression of PD-1 and PD-L1 both in tumor cells and immune infiltrates [52,60,65,66,70]. The second group concluded that expression of PD-1/PD-L1 is strictly correlated with grading, aggressive and metastatic disease. Patients in these studies also had a shorter survival time depending on the expression of molecules and the activity of the immune system [54,58,63]. Some studies included also less popular, yet key in immunological response, molecules. For example, explored the role of HHLA2 and B7x in GEP-NETs. Both proteins were expressed to a high degree in GI-NET and panNET tissues compared with adjacent non-neoplastic tissues. Furthermore, expression correlated with aggressive disease [49]. However, there are no approved molecules targeting this axis, and more in-depth research is needed, also in the context of combination therapy. Furthermore, hypoxia-inducible factor-α (HIFα) caused overexpression of B7x, and the blockade of the B7x molecule inhibited tumor cell proliferation and induced tumor cell apoptosis [49]. Moreover, the research showed that PD-L1 and B7-H3 were expressed in 53% and 78% NETs, respectively, and PD-L1 was expressed mainly in the cytoplasm. There was no association between PD-L1 and B7-H3 expression [69]. Interestingly, ref. [59] distinguished several types of GEP-NETs including the MLP-1 subtype with poorer prognosis and the highest expression of genes correlated with immunological responses. PD-L1 and PD-L2 were significantly highly expressed in MLP-1. Even though the results of the abovementioned studies are promising, a few limitations occurred. Importantly, different cut-off values and scoring systems are used in separate studies with different antibodies used in IHC, which leads to the incomparability of results.

### 3.2. Immune Cells Infiltrates

Immune cells in TME fall into two categories: adaptive immune cells and innate immune cells. Adaptive immunity with T cells, B cells, and natural killers (NK) cells is activated by exposure to specific antigens. On the contrary innate immunity is a non-specific defense mechanism against foreign antigens entering the body [24]. Anti-tumor cells are effector T-cells, including cytotoxic CD8+ T-cells and effector CD4+ T-cells, natural killer cells (NK), dendritic cells (DCs), and macrophages. Macrophages can be divided into two groups depending on their polarization, with M1 macrophages owning anti-tumor properties [87]. Tumor-promoting cells are Tregs and MDSCs. They can interplay with each other, affect anti-tumor cells, and coexist with tumor cells [87]. Among all the cells that populate the TME, immune infiltrates such as TILs, TAMs, dendritic cells, and mastocytes are the most abundant and are critically involved in cancer progression [88]. Furthermore, key interactions between immune cells and immune checkpoints are responsible for prognosis and patients’ responses to treatment, as mentioned before [25]. Multiple clinical trials confirmed the role of tumor-infiltrating immunological cells in response to immunotherapy. For example, pembrolizumab is one of the approved immunotherapies and has been successfully used in the therapy of patients diagnosed with pre-treated unresectable solid tumor with microsatellite instability (MSI-H) or mismatch repair deficiency (dMMR), due to its rich T-cell infiltration [89]. Studies including GEP-NETs also confirmed this thesis. Ref. [48] showed that the density of tumor-infiltrating lymphocytes inside the tumor appeared to be statistically significantly higher in highly malignant NENs than in low/medium malignant NENs (*p* < 0.001). In contrast, when it came to the density of these lymphocytes in the stroma, the difference was not significant. It was also shown that NENs with a high degree of malignancy had more PD-L1-positive lymphocytes than NENs with a low/medium degree of malignancy [48]. The research on immune cells in GEP-NEN G3 patients showed dense infiltration of PD-1+ CD8+ immune cells (the preferred cytotoxic cells in anti-tumor responses) only in PD-L1-positive areas of the tumor but not in PD-L1-negative areas. Furthermore, among patients with a large infiltration of CD8+ and PD-1+ cells, the median survival time was also longer (*p* = 0.04). It should be noted that patients defined as triple positive (PD-L1+, PD-1+, CD8+) showed the longest median survival compared to those that were triple negative (*p* < 0.01) [47]. Further studies also confirmed the previous results by defining the richer density of PD-1 positive TILs inside the tumor than the periphery. These cells also had higher PD-1/ICOS and PD-1/CTLA4 expression compared to healthy tissue, which could be a good target for double therapeutic blockade [70]. According to the study, a high density of CD206+ cells, a marker of tumor-associated macrophages, also correlated with prolonged survival [47]. Moreover, study [64] showed that a strong T-cell infiltration was associated with longer survival. Contradictory results showed an increased presence of CD3+, CD4+, CD8+, and FOXP3+ T lymphocytes, but no association was observed between lymphocyte density and prognosis [50]. According to [52], the immune environment of NETs of the pancreas and small intestine, the immunological characteristics of G3 tumors were not significantly different from their lower grade (G1, G2) counterparts. The infiltration of CD3+, CD45RO+, CD8+, and FOXP3+ cells (both effector and memory T-cells) was similar to that observed in lower-grade tumors. High levels of immune infiltration, defined by the presence of CD45RO+, CD3+, and CD8+ cells, were present in subgroups in both pancreatic and small bowel NETs but were generally more frequent in panNETs than in small intestine NETs (SI-NETs). The frequency of FOXP3+ (regulatory T cells) was relatively low in both panNET and SI-NET, indicating that regulatory T cells perhaps do not play a significant role in suppressing the immune response in this disease [52]. The immune infiltration of panNETs is characterized by considerable heterogeneity and includes CD4+, CD8+ T cells, and macrophages, among others [51,55,56,69]. In panNET tumors, a relatively lower expression of CD47 (interacts with signal regulatory proteins (SIRPα) on phagocytes such as macrophages and sends elimination evasion information) in tumor cells or higher numbers of CD163+ tumor-associated macrophages have been associated with poor prognosis, suggesting that these factors may act as prognostic indicators of panNET. In a healthy pancreas, CD47 was undetectable in the lobular and ductal cells but was observed in all panNET tumors. It is noteworthy that no correlation was shown between tumor grade (G1, G2, G3) and CD47 expression and CD163+ macrophages; however, the authors did not detail subpopulations of the macrophages [51]. In addition to the previously mentioned immune cells, there is also an increased infiltration of mast cells (they regulate the immune response and tumor formation) in neuroendocrine tumors. It was shown that the number of mast cells in panNENs with a low degree of malignancy was higher than in panNENs with an intermediate or high degree of malignancy, while the correlation between the number of macrophages and the degree of malignancy presented itself inversely. This study also found that CD3+ lymphocytes were the most abundant group of immune cells and that patients with a high mast cell density and a low number of these lymphocytes had a longer survival time without signs of disease progression [56]. Summarizing the aforementioned studies, the authors stated that rich immune-cell infiltration in TME correlates with rather an aggressive disease and poor prognosis. This is in contrast to many other studies, which showed that increased TILs were a prognostic factor for survival and predicted response to chemotherapy [90]. This may indicate that only a small, specific portion of GEP-NETs could benefit from immunotherapy as immunogenic tumors. However, a different conclusion was reached by Baretii et al., who showed that a higher number of CD3+ lymphocytes inside the tumor was associated with longer PFS [68]. The research showed that the density of CD8+ lymphocytes, PD-1+ lymphocytes, and CD 163+ macrophages was significantly higher in NEC than in other tissues [71]. Cai et al. noted that a high density of CD8+ T cells inside the tumor correlated with prolonged PFS, especially when the number of macrophages was low at the same time. In contrast, high densities of CD4+ lymphocytes and macrophages were associated with shorter median survival [69]. According to the retrospective analysis, the immune profile of the tissue is related to the histology of the tumor, with different groups observed for NETs and NECs. While NECs were characterized by a hot microenvironment with abundant lymphocytes infiltrating the tumor, NETs were characterized by a cold microenvironment with low lymphocyte density. Moreover, in NETs, the number of PD-1+ T lymphocytes and macrophages increased with the tumor grade [67]. Once again the strict correlation between tumor grade and immunological features was found; however, the results are contradictory when it comes to immune cells infiltrates, and more prospective studies are needed to define immunological answers in specific groups of GEP-NETs.

### 3.3. Cytokines

Considering the discovery of altered and dysregulated cytokine expression in all human cancers and the key role of cell communication in TME, cytokines have to be included in GEP-NETs analysis [91,92]. Not only are cytokines crucial in communication between cells in TME but also tumor cells secrete cytokines to suppress the immune response [93]. Among multiple specific cytokines, IL-6 has one of the best proven pro-inflammatory effects in the pathogenesis of cancer [94]. IL-6 is the crucial factor stimulating tumor cell proliferation, survival, and metastasis [95]. Mouse models showed that by stimulating cells in TME, IL-6 can foster immunosuppressive conditions, mostly by mediating cross-talk between tumor cells and activated tumor-infiltrating cells such as CAFs [96]. In lung cancer models, IL-6 promoted M2 polarization among macrophages, which led to elevated PD-1 mRNA expression in CD8+ T cells [97]. Furthermore, IL-6 showed an influence on myeloid-derived suppressor cells (MDSC), which promotes the progression of tumors by suppressing T-cell responses both in antigen-specific and nonspecific manners [95]. Moreover, it has been stated that IL-6 can interplay in immuno- and chemotherapy resistance in gastrointestinal cancers [98,99]. Therefore, it can serve as a negative prognostic marker and potential immunotherapy target [100]. Studies associated higher IL-6 expression in GEP-NETs with disease progression [57]. Moreover, elevated levels of IL-6 were associated with higher GEP-NET grade [62]. These results seem to confirm the potential role of IL-6 in GEP-NET’s pathogenesis and prognostic value for these neoplasms. The role of tumor necrosis factor-alpha (TNF-α) in cancer has been known for many years. In conclusion, constant production of this cytokine in TME has an anti-apoptotic effect, may mediate immune cell interactions, and induces a range of mechanisms that promote tumor development [101,102]. Interestingly, TNF- α may be a crucial factor in the metastatic process of multiple neoplasms [103]. Recent studies proved that TNF-α-mediated chronic inflammation can enhance epithelial cell acquisition of the mesenchymal phenotype, which leads to polarity and adhesion loss, resulting in the metastatic process [104]. The reviewed articles showed that TNF-α expression was commonly present in GEP-NETs and correlated with higher grading and was a negative prognostic marker [62]. Furthermore, authors accessed also other prevalent inflammation and cancer cytokines such as IL-1β, IL-2, and IFN-γ, which were overexpressed in TME [57,62]. Summing up, some cytokines can be used as a prognostic marker in GEP-NENs and by targeting the signaling they transduce, the effects of the immunotherapy can be increased [105].

### 3.4. Cancer-Associated Fibroblasts

Cancer-associated fibroblasts (CAFs) have been known for modulating the cancer microenvironment; their interaction with tumor cells results in tumor growth, angiogenesis, and invasion [106]. Additionally, CAFs have numerous interactions with tumor cells and the immune system. Through the secretion of multiple cytokines and other effector molecules such as IL-6, IDO, and TDO, CAFs directly modulate immune-cell-mediated antitumor immunity [107]. As mentioned before, CAFs also regulate immunosuppressive conditions in TME. Furthermore, CAFs induce the expression of PD-L1 on cancer cells, promoting tumor immune escape [108]. In GEP-NETs, elevated levels of TDO and IDO resulted from CAF activity [65]. TDO was expressed in 64% of tumors’ stromal cells, both enzymes created rich in the kynurenines microenvironment, which led to the suppression of effector T-cells or the conversion to tumor Tregs [65]. These results indicate that targeting CAFs in GEP-NENs can potentially reduce their immunosuppressive effect and improve the results of immunotherapy.

### 3.5. Neoangiogenesis

Advanced studies on neoangiogenesis in hypoxic conditions characteristic of tumors and other pathological states have been successfully conducted for years. Vascularization’s central role in the growth and spread of tumor cells to distant sites made it a potent therapy target and prognostic marker [109]. For instance, pancreatic cancer has been shown to have one of the strongest tumor neoangiogeneses with overexpression of vascular endothelial growth factor (VEGF) [110]. VEGF, as a main factor involved in both physiological and pathological angiogenesis, has been targeted in cancer treatment with effectiveness [111]. As for today, in hepatocellular carcinoma multikinase inhibitors, targeting the vascular endothelial growth factor receptor family (VEGFRs) is the standard of care. Multiple clinical trials and pre-clinical models showed efficacy in this implication [112,113]. Furthermore, studies showed that in some indications, immunotherapy can be applied by regulating TME vascularization and reversing the immunosuppressive effect of VEGF [114]. Furthermore, pre-clinical data concerning the VEGF role in GEP-NETs seems promising, as patients with metastatic disease had higher VEGF serum values when compared to patients without metastases (*p* = 0.033), and the highest levels were observed in the case of lymph node metastases (*p* = 0.008) [115]. Angiogenesis in GEP-NETs was accessed through the comparison of the neuroendocrine components of the tumor with non-neuroendocrine components, showing key differences between them. Neuroendocrine components had significantly higher expression of factors correlated with angiogenic activity such as vasohibin-1 (VASH-1) [71]. Furthermore, neoangiogenesis was correlated with dense CD163+ macrophages infiltration in neuroendocrine areas [71]. Comparing the high degree of vascularization of GEP-NENs and the associated infiltration of immune system cells, the use of angiogenesis inhibitors in combination with immunotherapy or chemotherapy may prove beneficial.

### 3.6. Microbiome

The pre-clinical studies established that there is a significant impact from the organism microbiome on cancer treatment, claiming that poor gut microbiota may worsen patients’ outcomes [116,117]. Commensal bacteria, in some manner, enhance the effect of chemotherapeutics on the tumor-infiltrating cells, and the administration of antibiotics shortly before chemotherapy weakened their effects [116]. Interestingly, research about the GEP-NENs TME and microbiome correlation has emerged. Among two groups of NENs, intestinal (I-NEN) and panNEN bacteria were common findings. Microorganisms were found in 75% of the I-NENs and 90% of the panNENs specimens. In the intestine, the distribution varied, yet in the pancreas, bacteria were found mainly in the proximity of blood vessels. However, no statistically significant differences were observed in mean bacterial count according to patients’ age, sex, ki 67% index, site, or tumor stage [72]. In conclusion, bacterial colonization has not been confirmed as a prognostic marker or pivotal player in tumor progression. However, limited data and a small study group suggest the possible existence of a crosstalk between intratumoral infiltrating bacteria and anti-tumor immunity and need future consideration.

## 4. Conclusions and Future Perspective

Taking everything into account, knowledge about NETs has improved in the last decade. Novel treatment options including immunotherapy have been presented for advanced tumors. Furthermore, treatment with immune checkpoint inhibitors can provide significantly improved response rates or even long-lasting complete responses in some cases [118,119,120]. Even though the success of immunotherapy has been proven in multiple clinical trials, to this day, a consensus regarding the optimal scope of patients for this therapy has not been reached [121,122,123,124]. TME is considered a key to the success of immunotherapy; however, the evaluation of the immune microenvironment and its clinical implications is still a challenge, especially in NENs. Recently, the clinical trials showed that immune checkpoint inhibitors have limited activity in GEP-NENs. Both phase Ib KEYNOTE-028 and the subsequent phase II KEYNOTE-158 investigating monotherapy with pembrolizumab showed an objective response rate (ORR) of 6.3% and 3.7%, respectively [125,126]. The phase Ib trial with anty-PD1 antibody toripalimab showed efficiency in NENs, especially in Pan-NETs with ORR 22.2%. The authors also established predictive markers—positive PD-L1 expression, tumor mutation burden, and microsatellite instability (MSI-H)—correlating with better responses [127]. Especially, the combined therapy targeting PD-1 and CTLA-4 showed activity in NECs. In the DART trial (NCT02834013), the objective responses were observed in NEC (ORR: 44%), with poor efficacy in the well-differentiated forms [128]. Moreover, numerous studies indicated severe immune-related (IR) adverse events (AEs) [129]. Practically every system can be affected by irAEs, including the gastrointestinal, pulmonary, endocrine, and cardiovascular systems. Effects range from mild to life-threatening, and their onset can be delayed several weeks or months [130]. The incidence of any-grade irAE in trials including patients with different solid tumor types was reported at 72% with ipilimumab and more than 50% with anti-PD-1/PD-L [131,132]. Moreover, fatal irAEs may occur; the trials reported toxicity-related fatality rates of 0.36% with anti–PD-1, 0.38% with anti–PD-L1, 1.08% with anti–CTLA-4, and 1.23% with combined anti–PD-1/anti–PD-L1 and CTLA-4 [133]. As was shown above, immunotherapy comes with significant consequences for the patient, may have limited activity, and often demonstrated no cost-effective approach to therapy [134]. In light of that, the key issue lies in finding a group of patients that has a potential chance of a favorable response with minimal side effects of the therapy. Based on the analysis of the TME, it is possible to recognize specific predictive factors that further decide on one’s qualification for efficient treatment [135,136,137]. Thus, the future research should be focused on NECs and G3 NETs and validating TME biomarkers, indicating an efficient response to PD-1/PD-L1 or CTLA-4 inhibitors.

At the current state, none of the investigated studies have presented an evident TME factor that may affect the clinical course of GEP-NETs. The results of the analyzed studies were usually contradictory. As the research has demonstrated, the GEP-NETs tumor microenvironment showed a close correlation with the tumor grade. Considering grading, G3 tumors showed dense infiltration of immune cells, which was often associated with the best prognosis. The most aggressive tumors had highly increased expression of immune checkpoints, presence of immune infiltration, and levels of pro-inflammatory cytokines. Furthermore, neoangiogenesis in GEP-NETs showed a strict correlation with inflammation and pro-inflammatory status in higher grades. Correlating the results of our analysis with the available results of the clinical trials, it seems that the greatest benefit from the use of immunotherapy can be achieved by patients with Pan-NECs. They are characterized by the richest immunological infiltration of TME, and at the same time, they perform best in clinical trials. However, the studies are inconsistent, and analytical methods regarding TME components arise to be a main challenge. For instance the lack of standardization for PD-L1 IHC in terms of the specificity and reproducibility of the available anti-PD-L1 antibodies and the subjective definition of the PD-L1 “positive” tumor. Furthermore, in the research, GEP-NETs are relatively rare neoplasms, even though clinical practice, and the personal observations of the authors show their increasing number. All of that limits the sample size and often includes patients treated according to different guidelines.

## Figures and Tables

**Table 1:** Characterization of the studies included in the review.

References	Authors	Samples	Technique	Neoplasm Grading	Study Group	Additional Information
[45]	Sampedro-Núñez et al.	formalin-fixed paraffin-embedded (FFPE) blocks	IHC (immunohistochemistry); IF (immunofluorescence)	G1; G2; G3	164	Analysis of PD-1 and PD-L1 expression in the TME, in addition to the characterization of the tumor immune cell, infiltrates.
[46]	Cives et al.	FFPE	IHC	G1; G2	102	Analysis of PD-1 and PD-L1 expression in the TME, in addition to the characterization of the tumor immune cell, infiltrates.
[47]	Rosery et al.	FFPE	IHC; mIF	G3; GEP-NECs	37	Analysis of PD-1 and PD-L1 expression in the TME. Focus on cytotoxic T-cells and TAMs.
[48]	Cho et al.	FFPE	Artificial intelligence (AI)-powered hematoxylin and eosin (H & E) analyzer	All	218	Analysis of TILs density and PD-L1 expression. Correlation between mentioned above factors.
[49]	Yuan et al.	Postoperative samples stored at −80 °C; samples from modified mice cohort	Multiple techniques for evaluating different markers: IHC; IF; RT-PCR; Western blot; among others	undefined	37 and additional mice cohort	Evaluation of expression of B7 immune-checkpoints: HERV-H LTR-Associating Protein 2 (HHLA2) and B7 Family Member X (B7x), hypoxia-inducible factor 1 alpha (HIF-1α).
[50]	Hiltunen et al.	FFPE	IHC	G1; G2	132	Analysis of the density of CD3+/CD8+/CD4+ and FOXP3+ T-cells.
[51]	Imam et al.	FFPE	IHC	G1; G2; G3	47	Analysis of the role of CD47 expression and CD163+ TAMs in panNETs
[52]	Da Silva et al.	FFPE	IHC; RNA sequencing	G1; G2; G3	95	Expression of PD-1; PD-L1 and PD-L2, profiling T-cell subsets in the TME. Additional RNA sequencing for further characterization of PD-L1 and PD-L2 expression.
[53]	Rösner et al.	FFPE	IHC	All	457	Evaluation of PD-1 and PD-L1 expression.
[54]	Roberts et al.	FFPE	IHC	GEP-NECs	37	PD-1 and PD-L1 expression in poorly differentiated NECs.
[55]	Wei et al.	FFPE; Fresh frozen NETs samples	IHC; RT-PCR; IF	G1; G2; G3	158	Analysis of TME with the determination of 14 immune signatures affecting patients’ prognoses.
[56]	Mo et al.	FFPE	IHC	All	187	Expression of CD117+ mast cells and CD68+ macrophages; CD15+ neutrophils; and CD3+, CD4+, and CD8+ T cells in panNENs.
[57]	Pereira et al.	FFPE	IHC	G1; G2	39	Expression of IL-6, Ki-67, FOXM1, and IGF1R in GEP-NETs.
[58]	Bösch et al.	FFPE	IHC	G1; G2; G3	244	Expression of PD-1/PD-L1 and characterization of TILs in GEP-NENs
[59]	Young et al.	Fresh frozen panNETs samples	RNA sequencing	G1; G2; G3	207	Analysis of expression of immune-related genes in panNETs
[60]	Busse et al.	FFPE	IHC; mRNA immunoprofiling	All	78	Expression of immune-related factors in the TME.
[61]	Sato et al.	FFPE	IHC	G1; G2; G3	16	Analysis of TILs and human leukocyte antigen (HLA) class I and other factors.
[62]	Herman Mahečić et al.	FFPE	IHC	G1; G2; G3	43	Analysis of the role of tumor necrosis factor alpha (TNF-α), interleukin 1 beta (IL-1β), IL-2, and IL-6 in GEP-NENs
[63]	Milione et al.	FFPE	IHC	All	315	Analysis of immune-, inflammatory-, angiogenesis-, proliferation-, and fibroblast-related biomarkers
[64]	Centozone et al.	FFPE	IHC	G3; NECs	45	Analysis of myeloid markers—CD33, CD163, and Arginase in High-Grade GEP-NETs.
[65]	De Hosson et al.	FFPE	IHC	G1; G2	41	Analysis of PD-L1, T-cells, indoleamine 2,3-dioxygenase (IDO) and tryptophan 2,3-dioxygenase (TDO), mismatch repair proteins (MMRp), and activated fibroblasts.
[66]	Ali et al.	FFPE	IHC	G3	136	Analysis of expression of PD-L1 in G3 GEP-NENs.
[67]	Takahashi et al.	FFPE	IHC with multispectral imaging	All	52	Analysis of TILs, macrophages, and PD-1/PD-L1 expression.
[68]	Baretii et al.	FFPE	IHC	undefined	36	Analysis of CD3, CD8, PD-1, PD-L1, IDO expression
[69]	Cai et al.	FFPE	IHC	G1; G2	104	Analysis of TAMs and HLA-I/II, PD-L1, B7-H3 expression
[70]	Vesely et al.	FFPE; fresh frozen NETs samples	IHC; FCM	G1; G2; G3	40	Analysis of T-cell subsets in the TME, characterization of expression of immune checkpoint molecules.
[71]	Tsunokake et al.	FFPE	IHC	NECs	33	Analysis of immune microenvironment in addition to comparing TILs, TAMs, and other relevant factors in the components of the same tumor.
[72]	Massironi et al.	FFPE	fluorescent in situ hybridization (FISH) by confocal microscopy	G1; G2; G3	40	Analysis of GEP-NENs microbiome and its correlation with the immune microenvironment.

**Table 2:** Key findings of the selected studies.

References	Key Findings
[45]	PD-1/PD-L1 were expressed in 1 to 8% of GEP-NEs and can be correlated with disease progression.
[46]	Expression of PD-L1 was higher in duodenal NETs than in ileal/jejunal. One-third of tumors were immunologically ignorant and unsuitable for immune checkpoint blockade.
[47]	Intense PD-1+ CD8+ immune cell infiltration showed the most favorable median overall survival (OS).
[48]	TIL density and PD-L1 expression were both significantly higher in high-grade NENs.
[49]	Higher expression of B7x and HHLA2 correlated with higher grade and higher incidence of nodal and distal spread. Furthermore, expression of the above factors was correlated with hypoxia and HIF-1α.
[50]	There was no correlation between CD3+, CD4+, CD8+, and FOXP3+ T-cells density in TME and patients’ prognosis.
[51]	CD47 was overexpressed in panNETs; moreover, CD163+ TAMs were correlated with higher grade and distal spread.
[52]	No significant difference in the PD-1, PD-L1, and T-cell infiltrate levels was spotted between G1, G2, and G3 tumors. Expression of immune checkpoints was rare in GEP-NETs.
[53]	PD-L1 expression was common in GEP-NENs and increased with grading.
[54]	PD-1 and PD-L1 expression was a common event in poorly differentiated NECs.
[55]	T-cells and macrophages were dominant infiltrates in panNETs, CCL19, IL-16, CD163, IRF4, and CD8 and can be possible predictors of immune responses.
[56]	CD117+ mast cells showed a protective role in panNENs. High mast cell infiltration was correlated with elevated CD4+ T-cells.
[57]	IL-6 expression in GEP-NETs can be correlated with disease progression. Furthermore, patients with low HDL cholesterol expression had higher IL-6 peritumoral expression.
[58]	High TILs and PD-1 expression were significantly associated with shorter patient survival and higher grading in GEP-NENs. PD-L1 expression showed a trend of shorter patient survival.
[59]	Detailed information about molecular subtypes: metastasis-like primary MLP-1 and MLP-2, insulinoma-like and intermediate. MLP-1 subtype correlated with higher immune-related genes expression and immune responses in TME.
[60]	G1/G2 NENs differ from poorly differentiated NENs. Both NET G1/G2 and NET G3/NEC showed low expression of IFNγ-associated genes and low intratumoral T-cell infiltration.
[61]	CD4+, CD8+, and CD45RO+ (memory) T-cells were present in TME; simultaneously, there was no correlation between TILs and patients’ prognosis.
[62]	High expression of TNF-α was correlated with higher tumor grades. GEP-NENs had higher expression of IL-6 than IL-1β or IL-2.
[63]	G1/G2 versus G3 GEP-NENs showed divergence with immune-inflammatory markers. G1/G2 to G3 transition was associated with immune-inflammatory profile changes.
[64]	High-grade NENs could be divided into prognostic sub-groups based on myeloid and T-cell markers. Tumors with a high density of the abovementioned markers show a better prognosis.
[65]	Expression of factors correlated with immune checkpoint treatment responses were present to a limited extent or even absent. TDO and IDO were expressed in more than 50% of NETs.
[66]	PD-L1 expression was present in only a small subset of G3 tumors. This factor shows no correlation with clinical parameters and prognosis.
[67]	While NECs can be characterized by hot immune microenvironments with abundant TILs, NETs had a cold immune microenvironment with few TILs. Several intraepithelial PD-1+ T-cells and PD-L1+ macrophages were elevated according to the grade.
[68]	Higher intratumoral CD3+ T-cell infiltrate was associated with a better prognosis. Expression for CD3/8, IDO, and PD-1 differed among the interface and the tumor.
[69]	The high amount of CD8+ T-cell infiltration with low TAMs can be correlated with a positive prognosis.
[70]	TILs were present in less than 10% of tumors, however intratumoral TILs had higher expression of PD-1. Moreover, CD8+ TILs had higher expression of PD-1 and CTLA-4.
[71]	Comparing neuroendocrine and non-neuroendocrine areas, there was more angiogenic activity and a more suppressive microenvironment in neuroendocrine areas.
[72]	Ninety percent of NETs showed microorganisms’ infiltration, with a homogeneous microbial distribution. Bacterial localization in panNEN was observed in the proximity of blood vessels.

## Data Availability

The data can be shared upon request.

## Abbreviations

NEN	neuroendocrine neoplasm
NET	neuroendocrine tumor
NEC	neuroendocrine carcinoma
GEP-NET	gastrointestinal neuroendocrine tumor
panNET	pancreatic neuroendocrine tumor
SI-NETs	small intestine neuroendocrine tumor
TME	tumor microenvironment
CT	computed tomography
MRI	magnetic resonance imaging
EUS	endoscopic ultrasound
SSA	somatostatin analog
PET	positron emission tomography
mTOR	mammalian target of rapamycin
TKI	tyrosine kinase inhibitor
IHC	immunohistochemistry
IF	immunofluorescence
FFPE	formalin-fixed paraffin-embedded
PD-1	programmed cell death-1
PD-L1	programmed cell death-1 ligand
SHP-2	homology region 2 domain-containing phosphatase (SHP-2)
TAM	tumor-associated macrophages
TIL	tumor-infiltrating lymphocyte
RT-PCR	reverse transcription polymerase chain reaction
HHLA2	human endogenous retrovirus H long terminal repeat-associating 2
B7x	B7 homolog x
HIF-1α	hypoxia-inducible factor 1 alpha
FOXM1	Forkhead box protein M1
IGF1R	type 1 insulin-like growth factor receptor
HLA	human leukocyte antigen
TNF-α	tumor necrosis factor alpha
VEGF	vascular endothelial growth factor
IDO	indoleamine 2,3-dioxygenase
TDO	tryptophan 2,3-dioxygenase
B7-H3	B7 homolog 3 protein
OS	overall survival
PFS	progression-free survival.
CTLA-4	cytotoxic T-lymphocyte-associated protein-4
CAF	cancer-associated fibroblast
VASH-1	vasohibin-1
MDSC	myeloid-derived suppressor cells
FCM	flow cytometry
irAEs	immune-related adverse events
